# Diagnosing Nodular Regenerative Hyperplasia of the Liver Is Thwarted by Low Interobserver Agreement

**DOI:** 10.1371/journal.pone.0120299

**Published:** 2015-06-08

**Authors:** Bindia Jharap, Dirk P. van Asseldonk, Nanne K. H. de Boer, Pierre Bedossa, Joachim Diebold, A. Mieke Jonker, Emmanuelle Leteurtre, Joanne Verheij, Dominique Wendum, Fritz Wrba, Pieter E. Zondervan, Jean-Frédéric Colombel, Walter Reinisch, Chris J. J. Mulder, Elisabeth Bloemena, Adriaan A. van Bodegraven, NRH-pathology Investigators

**Affiliations:** 1 Department of Gastroenterology and Hepatology, VU University Medical Center, Amsterdam, the Netherlands; 2 Department of Pathology, Beaujon Hospital, Paris, France; 3 Department of Pathology, Cantonal Hospital, Lucerne, Switzerland; 4 Department of Pathology, Refaja Hospital, Stadskanaal, the Netherlands; 5 Department of Pathology, CHRU Lille, Lille, France; 6 Department of Pathology, Academic Medical Center, Amsterdam, the Netherlands; 7 Department of Pathology, Saint-Antoine Hospital, Paris, France; 8 Department of Pathology, University Medical Center Vienna, Vienna, Austria; 9 Department of Pathology, Erasmus Medical Center, Rotterdam, the Netherlands; 10 Department of Gastroenterology, Icahn School of Medicine at Mount Sinai, New York, New York, United States of America; 11 Department of Gastroenterology and Hepatology, University Medical Center Vienna, Vienna, Austria; 12 Department of Pathology, VU University Medical Center, Amsterdam, the Netherlands; 13 Department of Internal Medicine, Gastroenterology and Geriatrics, ORBIS Medical Center, Sittard-Geleen, the Netherlands; Icahn School of Medicine at Mount Sinai, UNITED STATES

## Abstract

**Background and Aims:**

Nodular regenerative hyperplasia (NRH) of the liver is associated with several diseases and drugs. Clinical symptoms of NRH may vary from absence of symptoms to full-blown (non-cirrhotic) portal hypertension. However, diagnosing NRH is challenging. The objective of this study was to determine inter- and intraobserver agreement on the histopathologic diagnosis of NRH.

**Methods:**

Liver specimens (n=48) previously diagnosed as NRH, were reviewed for the presence of NRH by seven pathologists without prior knowledge of the original diagnosis or clinical background. The majority of the liver specimens were from thiopurine using inflammatory bowel disease patients. Histopathologic features contributing to NRH were also assessed. Criteria for NRH were modified by consensus and subsequently validated. Interobserver agreement was evaluated by using the standard kappa index.

**Results:**

After review, definite NRH, inconclusive NRH and no NRH were found in 35% (23-40%), 21% (13-27%) and 44% (38-56%), respectively (median, IQR). The median interobserver agreement for NRH was poor (κ = 0.20, IQR 0.14-0.28). The intraobserver variability on NRH ranged between 14% and 71%. After modification of the criteria and exclusion of biopsies with technical shortcomings, the interobserver agreement on the diagnosis NRH was fair (κ = 0.45).

**Conclusions:**

The interobserver agreement on the histopathologic diagnosis of NRH was poor, even when assessed by well-experienced liver pathologists. Modification of the criteria of NRH based on consensus effort and exclusion of biopsies of poor quality led to a fairly increased interobserver agreement. The main conclusion of this study is that NRH is a clinicopathologic diagnosis that cannot reliably be based on histopathology alone.

## Introduction

Nodular regenerative hyperplasia (NRH) is a vascular disorder of the liver which is increasingly recognized in daily clinical practice. The occurrence of NRH is associated with certain diseases, including immune mediated disorders, such as inflammatory bowel disease (IBD). Nodular Regenerative Hyperplasia in HIV-infected patients seems strongly associated with the cumulative exposure to didanosine and stavudine, anti-retroviral drugs causing toxic injury to sinusoidal endothelial cells. [1–8]

Nodular Regenerative Hyperplasia has been reported to be an underrated adverse event of thiopurine use, widely prescribed in IBD patients. [6–10] The clinical manifestation of NRH consists of symptoms of non-cirrhotic portal hypertension, such as splenomegaly, thrombocytopenia and oesophageal varices. [11,12] NRH is allegedly associated with the development of hepatocellular carcinoma. [13]

Although mostly referred to as a clinical disease entity, NRH is actually a histopathologic feature that generally coincides with other histopathologic features indicative of vascular liver toxicity such as phlebosclerosis (obliterative portal venopathy), sinusoidal dilatation, sinusoidal obstruction syndrome, centrilobular venopathy and perisinusoidal fibrosis. [12,14]

The clinical significance of a histopathologic diagnosis of NRH is yet unclear, as NRH associated symptoms are diverging from absence of symptoms or mild liver test abnormalities, up to severe complications such as portal hypertension. [6,7]

The temporal relation between development of histopathologic NRH and clinical signs of portal hypertension is not yet elucidated. Whether non-cirrhotic portal hypertension is the obligatory clinical end-stage in all patients diagnosed with NRH or only in a subset has not been established either. [15,16]

The discussion concerning the histopathologic diagnosis of NRH was revived due to its association with tioguanine and conventional thiopurine use in IBD patients. [6–8,17] Based on this association, the ongoing clinical use of thiopurines, in particular tioguanine, has been strongly discouraged ever since.

However, the diagnosis of NRH may be challenging due to different interpretations of specific histopathologic features by pathologists. Therefore, in 1990, Wanless redefined the definition of NRH as part of a new classification of micronodular transformation. [18] These criteria have never been validated. The primary objective of this study was to calculate the inter- and intraobserver agreement for the histopathologic diagnosis of NRH and the specific characteristics that contribute to this diagnosis, focusing on patients with IBD who were treated with thiopurines.

## Material and Methods

### Study design

**Fig 1:**
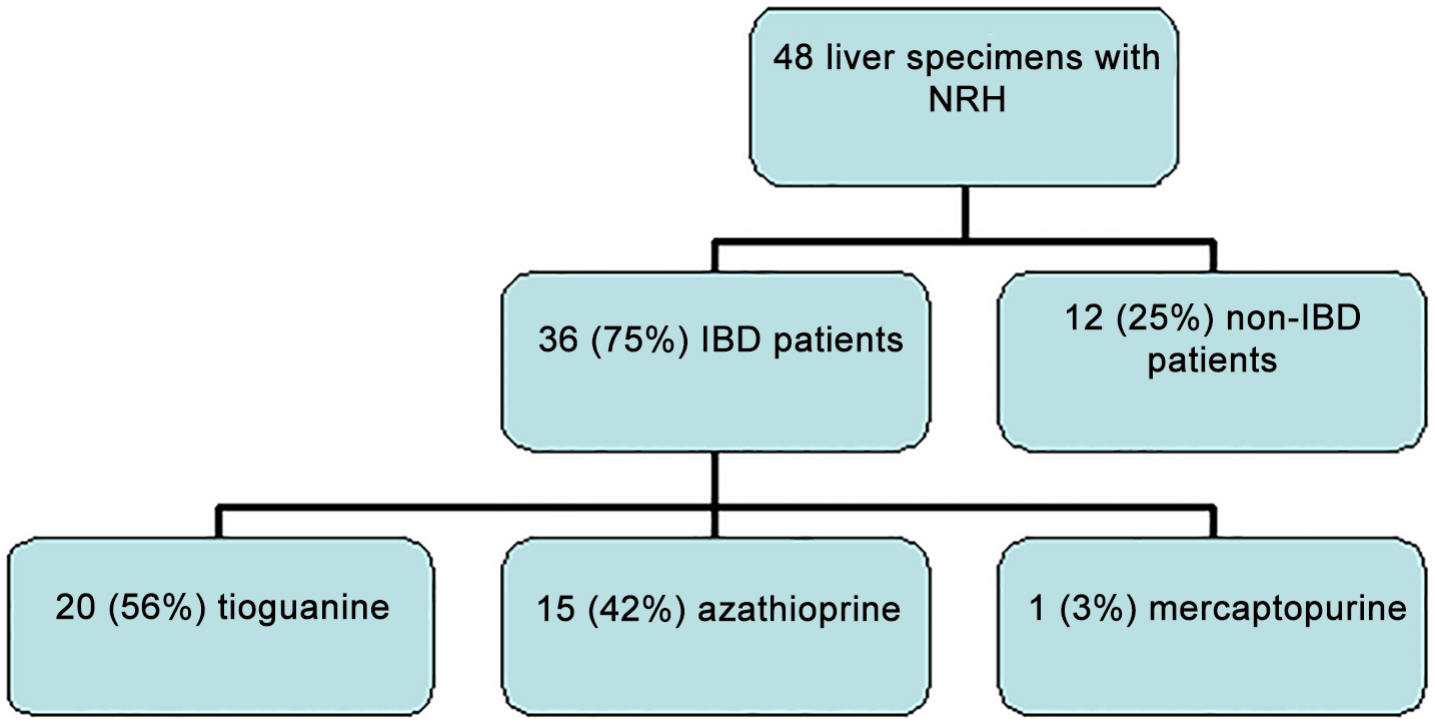
Origin of liver specimens with Nodular Regenerative Hyperplasia (NRH).

From six different European third-line referral hospitals (Munich, Vienna, Rotterdam, Paris, Lille and Amsterdam), 48 liver biopsy specimens previously diagnosed solely as NRH were collected. Thirty-six liver specimens were obtained from IBD patients who had been using azathioprine, mercaptopurine or tioguanine. Twelve liver biopsies came from non-IBD patients who had no (previous) exposure to thiopurines (Fig 1).

Thirty-four out of the 36 specimens that were obtained from thiopurine exposed IBD patients were previously reported in three studies that addressed the safety of either azathioprine or tioguanine treatment in IBD. [7,19,20] All liver specimens were individually reviewed by seven experienced liver pathologists, six of whom were affiliated with the hospital where the biopsy specimens were obtained. The pathologists were not provided with clinical information nor were they aware that only biopsy specimens previously diagnosed as NRH were included in the review process. Five out of seven pathologists additionally scored specific histologic features that contribute to the histopathologic diagnosis of NRH including characteristics of nodularity and fibrosis. Two pathologists also assessed the quality of the biopsy specimens.

After independent examination of the liver slides, all pathologists attended a meeting held at the VU University Medical Center in Amsterdam, the Netherlands, to develop an updated consensus on the existing histopathologic diagnosis of NRH and its specific features. Liver slides were collectively reviewed to provide a definitive and unanimous diagnosis, being used as the histopathologic gold standard for validation of the consensus. The updated histologic criteria from the consensus meeting were independently validated by two experienced liver pathologists, who were not involved in the earlier consensus meeting.

### Ethical considerations

The liver specimens that were not obtained in the Netherlands and were primarily obtained for the purpose of other studies, were obtained after written informed consent in the two studies reporting on the use of tioguanine. [19,20] The biopsy specimens from the third study, obtained in an azathioprine using cohort, as well as the remaining liver specimens included in this study were performed as part of standard care; verbal consent was obtained. [7] All liver specimens were sent to Amsterdam without any identifying information. As patients included in this study were not subjected to additional tests or questionnaires no official ethical approval was required according to the Dutch Medical Research Involving Human Subjects Act. According to the Dutch code of conduct for dealing responsibly with human tissue in the context of health research, all specimens and clinical data used in this study were coded anonymous.

### Liver biopsy specimen

Liver specimens were obtained by percutaneous needle biopsy or by surgical wedge resection during a surgical procedure indicated for the treatment of IBD. All specimens had been stained with hematoxylin & eosin (H&E), reticulin and at least one collagen stain (Masson’s trichrome, Sirius red stain or Elastica von Giesson stain). The quality of liver specimens was assessed by evaluating total biopsy length, the number of portal tracts and whether the biopsy specimen was fragmented.

### Study outcome

The primary outcome of the study was to evaluate inter- and intraobserver agreement on the diagnosis of NRH. All pathologists were familiar with the criteria for NRH as proposed by Wanless: NRH was present if nodularity was distinct in most areas examined in both the H&E and reticulin staining (Grade 3). Septal fibrosis had to be either absent (Grade 0), or only occasionally present but not surrounding the nodules (Grade 1) to be compatible with NRH. [17] In this study, the presence of NRH was subsequently scored as: definite NRH, no NRH or inconclusive NRH if the pathologists believed that technical shortcomings hampered a proper evaluation.

Five out of seven pathologists assessed specific histopathologic features that characterize NRH. These features included the presence of hepatocellular nodules < 3mm, regions of hyperplasia with a curved contour, regions of atrophy surrounding the regions of hyperplasia, liver cell plates > 1 cell layer thick producing a compression zones at the periphery of nodules and (the absence of) septal fibrosis.

### Statistical analysis

Continuous variables were expressed as the median with interquartile range (IQR). Categorical variables were expressed as numbers and percentages. Intraobserver variation was calculated as the percentage of NRH diagnosed by each pathologist compared with the original 100 percent score on biopsies assessed by the same pathologist. The interobserver agreement was evaluated by calculating the kappa index (κ), which excludes chance-expected agreement. The kappa could range from <0.4 = poor; 0.4–0.75 = fair to good; >0.75 = excellent. Kappa values were calculated for comparisons between each pair of pathologists. The overall interobserver agreement kappa between all pathologists was calculated for NRH and the specific features of NRH. To evaluate the potential influence of the quality of specimens, a sub-analysis with regard to interobserver agreement was made by selecting high quality liver slides (i.e. larger than 10mm and without technical shortcomings).

The observer bias was also investigated. [21] An observer bias means that disagreement between observers is not attributable to chance, and that there is a significant trend for overestimation or underestimation by certain observers. A *p*-value less than 0.05 was considered to be statistically significant. Statistical analysis was performed using the Statistical Package for the Social Sciences version 16.0, Chicago, USA.

## Results

### Biopsy specimens and patient characteristics

Forty-eight liver specimens were examined in this study. Indications for liver biopsy were signs of portal hypertension and/or biochemical liver abnormalities, use of tioguanine or unknown in respectively 46%, 42% and 13%. Biopsy specimens had a median length of 20 mm (IQR 13–26 mm). A significant part of patients suffered from signs not specifically for, but indicative of, portal hypertension such as oesophageal varices, thrombocytopenia, splenomegaly, hepatofugal flow and ascites (Table 1).

**Table 1:** Patient characteristics including clinical symptoms of portal hypertension.

Total number of liver specimens = 48	
**IBD/ non-IBD**	36/ 12
**Thiopurines/ no Thiopurines**	36/ 12
**Thiopurines (n = 36)**	
Azathioprine (median dosage)	15 (2 mg/kg/day, IQR 2.0–2.25)
Mercaptopurine	1 (1 mg/kg/day)
Tioguanine (median dosage)	20 (40 mg/day, IQR 40–40)
**Portal hypertension (endoscopy and/or liver imaging)**	**Percentage**
Hepatofugal flow	24% (9/37)
Splenomegaly	31% (13/42)
Collaterals	17% (7/42)
Ascites	7% (3/41)
Oesophageal varices	44% (14/32)
Any of above mentioned symptoms	
	50% (21/42)

IBD, inflammatory bowel disease.

Poor intra- and interobserver agreement on the diagnosis of NRH and its specific histopathologic criteria.

Re-assessment of the biopsy specimens resulted in large intra- and interobserver differences. The intraobserver agreement on definite NRH ranged between 14% and 71%, with a median of 67% (Table 2).

**Table 2:** Intra and inter observer variability of nodular regenerative hyperplasia.

Pathologist	1	2	3	4	5	6	7
**1**	***67%***	0%	0%	67%	67%	33%	100%
**2**	64%	***64%***	18%	46%	55%	27%	55%
**3**	14%	7%	***14%***	14%	14%	7%	14%
**4**	50%	0%	0%	***67%***	0%	33%	33%
**5**	29%	43%	0%	29%	***71%***	71%	43%
**6**	29%	0%	29%	29%	57%	***71%***	43%

Intraobserver (bold, italic cells) and interobserver variability in the diagnosis of NRH after re-assessment of liver slides by seven pathologists. Six pathologists from six hospitals provided liver slides that had originally been diagnosed as NRH. Pathologist 1 corresponds with pathologist 1, etc. Pathologist 7 did not provide liver slides for this study.

Between pathologists, variation in the diagnosis of definite NRH ranged from 13% to 40%, with a median of 35%. Diagnosis of inconclusive NRH, and no NRH ranged from 13% to 40% (median 21%) and 21% to 75% (median 44%), respectively (Table 3).

**Table 3:** Diagnosis of nodular regenerative hyperplasia on re-examination of all 48 liver specimens.

Pathologist	1	2	3	4	5	6	7
**Definite NRH**	38%	23%	13%	35%	40%	35%	40%
**Inconclusive NRH**	19%	21%	13%	27%	40%	21%	13%
**No NRH**	44%	56%	75%	38%	21%	44%	48%

Some pathologists did not observe any NRH in the liver slides provided by other medical institutes. The median interobserver agreement for the histopathologic diagnosis of NRH, scored by all seven pathologists, was poor (κ = 0.20, IQR 0.14–0.28). The intra-observer agreement of one of the pathologists was very low (14%). After exlusion of this pathologist, inter-observer agreement for NRH remained poor (κ = 0.23, IQR 0.15–0.30).

The median interobserver agreement for definite NRH was not statistically significant different between IBD and non-IBD patients. Twenty-four out of 48 (50%) liver biopsies, including all surgical wedge resections (n = 5), were arbitrarily considered adequate for proper evaluation as these liver biopsies were more than 10 mm in length and were not fragmented. Interobserver agreement, however, remained similar with restricted use of only these adequate liver specimens (data not shown).

Five pathologists additionally assessed the specific histolopathologic features of NRH. The median interobserver agreement for characteristics of NRH were 0.31 for hepatocellular nodules < 3mm (IQR 0.17–0.38), 0.17 for regions of hyperplasia (IQR 0.06–0.22), 0.25 for regions of atrophy (IQR 0.20–0.34), 0.11 for compression zones (IQR 0.06–0.35) and 0.49 for absence of septal fibrosis (IQR 0.41–0.58).

### Consensus view on the specific characteristics of NRH and proposed modified definition

Collective reassessment of liver slides by the participating pathologists resulted in a slight modification of the histopathologic definition of NRH as proposed by Wanless in 1990. [18] This adapted consensus view on the diagnosis of NRH did acknowledge that for a histopathologic diagnosis of NRH the presence of grade 3 micronodularity (distinct nodularity in both H&E and reticulin staining) was mandatory. In contrast to Wanless’ definition in which grade 1 septal fibrosis was accepted, the consensus view was that any septal fibrosis excluded a diagnosis of NRH. The recognition of (non-septal) perisinusoidal fibrosis, however, was added and was found to be compatible with NRH. In addition, a description of the extent of nodularity (i.e. focal or diffuse) complemented the description of the biopsy. In case of severe steatosis (more than 2/3 of liver biopsy) or steatohepatitis, the adapted consensus view was that NRH could not be properly assessed. The quality of liver specimens was believed to be of great importance for properly diagnosing NRH. Therefore, both specimen length and whether or not the specimen was fragmented should be specified. Despite technical shortcomings such as short length and fragmentation, a histopathologic diagnosis of NRH can be made. However, it was believed by consensus that technical shortcomings of a biopsy specimen could not exclude the presence of NRH (Table 4).

**Table 4:** Description of criteria compatible with nodular regenerative hyperplasia (NRH) of the liver according to Wanless et al.^[^18^]^ versus Jharap et al.

NRH	Wanless et al.^[^ ^18^ ^]^	Jharap et al.
Hepatocellular nodules	Regions of atrophy juxtaposed to normal or hyperplastic regions with a curved contour	Central part of enlarged hepatocytes and/or thickened liver cell plates with a rim of smaller hepatocytes and/or thinner liver cell plates at the outer border of the nodule with compression of the sinuses in the periphery
Fibrous septa	Hepatocellular nodules are not surrounded by fibrous septa	Hepatocellular nodules are not surrounded by fibrous septa
Perisinusoidal fibrosis	Occasional septa not surrounding nodules is compatible with NRH	Perisinusoidal or pericellular collagen fibrosis is compatible with NRH
Cell plates	In NRH, hepatocytes are often arranged in double-cell plates	In NRH, hepatocytes are often arranged in double-cell plates

**Fig 2:**
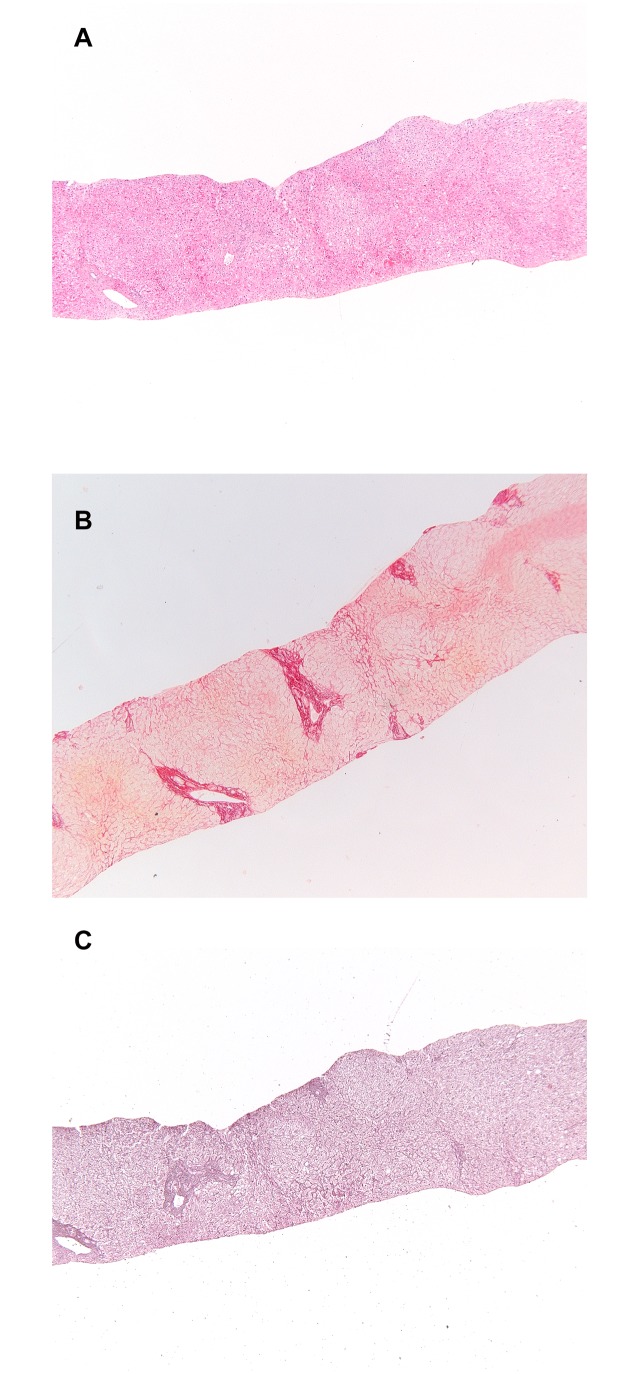
Histopathologic liver specimen with nodular regenerative hyperplasia. A. Hematoxylin & eosin staining. B. Reticulin staining in which the nodules are apparent. C. Sirius red staining highlighting the subtle collagen fibres around the atrophic liver cell plates.

These subtle changes to the definition of NRH led to the following description: nodular regenerative hyperplasia of the liver is characterized by the focal or diffuse appearance of hepatocellular nodule(s) less than 3mm in diameter consisting of a central part of enlarged hepatocytes and/or thickened liver cell plates with a rim of smaller hepatocytes and/or thinner liver cell plates with compression of the sinuses in the periphery where perisinusoidal but not septal fibrosis may occur. The nodules need to be distinct on both H&E and reticulin staining (Fig 2).

### Portal hypertension in definite NRH, inconclusive NRH and no NRH

During the consensus meeting, 32/48 liver biopsies were collectively assessed and consensus was reached. NRH, inconclusive NRH and no NRH were diagnosed in 7/32 (22%), 4/32 (13%) and 21/32 (66%) liver biopsies, respectively. Portal hypertension was reported in 71%, 50% and 38% of the patients with respectively definite NRH, uncertain NRH and no NRH.

### Calculation of observer agreement when using the modified definition of NRH

The updated description of the consensus definition of NRH was used for validation by two independent liver pathologists, who did not participate in the earlier phases of this study. The update of the definition of NRH resulted in a slight increase of the interobserver agreement (kappa = 0.35), however this still remained poor. The interobserver agreement further improved (kappa = 0.45) after exclusion of liver biopsies with technical shortcomings.

## Discussion

This study showed that the interobserver agreement of NRH and specific histologic features of NRH in properly stained liver biopsies was poor, even when assessed by well-experienced liver pathologists. Moreover, large differences in intraobserver agreement were observed after reassessment of liver slides, previously diagnosed with NRH. Refining existing criteria based on a consensus effort led to only a marginal improvement.

The main strength of this study was its multi-center character involving a large group of liver pathologists from all over Europe. Of importance, clinical data, such as that the majority of the included liver specimens were from IBD patients on past or present thiopurine use, were being withheld from the pathologists, aiming at unbiased (microscopy-based) scoring of the liver biopsy specimens. Furthermore, there seemed no major role for a potential observer bias as the intra- and interobserver agreements were poor even though all pathologists knew they were participating in a study on NRH.

There are also some limitations of our study. First, although initially designed to calculate interobserver agreement on an established diagnosis of NRH, the low agreement between and within observers (pathologists) raised the question whether addition of a series of control biopsy specimens would have improved diagnostic accuracy. The ‘true” diagnostic accuracy of histologic readings, measured by the traditional indices of sensitivity, specificity, positive and negative predictive value cannot be determined from this study in the absence of both negative controls and a gold standard for positive liver biopsies. In this regard however, it is essential to mention that our study did not intend to assess diagnostic accuracy, but was designed instead to explore inter- and intraobserver agreements, neither of which by definition requires control groups nor gold standard criteria for positive liver biopsies. Until now, the diagnosis of NRH was based on criteria described by Wanless, even though these criteria have never been validated. [18] Our study demonstrated that the variability of the histologic diagnosis of NRH, based on the Wanless criteria, is high, therefore a gold standard for NRH does not exist, i.e. a positive control group is not possible. In addition, the aim of the study focused on the reproducibility of the histologic diagnosis of NRH. A negative control group was not added, as it is very challenging to define a negative control group, as several studies have shown that there is already a significant interobserver variability in assessment of liver biopsies with regard to other diagnoses besides NRH. [22]

Secondly, the quality of several biopsies used in this study was scored as being of suboptimal quality for microscopic examination. Approximately half of the biopsies were fragmented and had a total length of less than 10mm. This, however, reflects day-by-day clinical practice both regarding the harvest of liver biopsy specimens as well as the material which is provided to pathologists to make a histopathologic diagnosis. Rousselet et al. showed that quality of the liver biopsy may influence inter-observer agreement. [21] Nevertheless, the exclusion of small and fragmented biopsies from our study only led to a marginal increase in interobserver agreement from 0.35 to 0.45. This discrepancy may be due to small sample size.

Finally, this study was only designed to specifically address the histopathologic diagnosis of NRH. The participating pathologists were not aware of the clinical background of the patients when they reviewed the liver slides; therefore there was no bias during re-assessment of the liver slides. However, the majority of patients had a history of thiopurine use, which may have caused patient selection bias. From a more clinical perspective, it would have been of additional interest to explore other histopathologic features, such as sinusoidal dilatation, dilated portal veins (shunt vessels), phlebosclerosis, centrilobular fibrosis, portal tract remnants and perisinusoidal fibrosis, which are also associated with idiopathic non-cirrhotic portal hypertension. [3,12,23] This may be considered as a limitation.

The reasons for the observed low inter-observer agreement on the histopathologic diagnosis of NRH even after modification of its definition are uncertain. It is fair to mention that following the consensus, the definition of NRH was only slightly modified as compared to the definition of Wanless. [18] Hence, this definition can still be subject to different interpretation. There are several possibilities for improvement of the poor inter-observer agreement of NRH. It has been shown that education or consensus reading may result in improvement of inter-observer agreement. [21] Although the involved pathologists used the updated description of the consensus definition for NRH during validation, the kappa of NRH didn’t improve significantly, indicating that consensus reading for the diagnosis NRH is not sufficient enough, and pathologists need to be educated in the updated criteria for NRH. Rousselet et al. showed that quality of the liver biopsy, such as length may influence inter-observer agreement. [21] Our study didn’t demonstrate improvement of the kappa for NRH after exclusion of inadequate liver biopsies, which may be explained by small sample size. Although the lack of clinical information may have resulted in unbiased scoring of liver biopsies, it could also explain the remarkable discrepancy between the proportion of NRH diagnosed at the original examination when clinical data were available, and the lower proportion of NRH after reassessment of the same liver biopsies, when patient characteristics were not provided. Unfortunately, our study didn’t investigate the influence of clinical data or pathologists’ experiences and training on the interobserver agreement. Future studies are needed to demonstrate whether adding clinical information improves inter-observer agreement.

Additionally, the diagnosis of NRH is not always obvious at liver biopsy and NRH can present itself in different histologic grades. However, the diagnosis NRH is highly likely, when histologic criteria of NRH are present in a liver biopsy of a patient using NRH-associated drugs and presenting himself with clinical signs of non-cirrhotic portal hypertension. Interpretation of literature regarding prevalence or incident cases of drug or disease induced NRH appears to be thwarted by the incongruence between histopathology and clinical syndrome as well. Ideally non-invasive diagnostic tests would replace the liver biopsy in the future. Efforts have been made to diagnose NRH by MRI and by measuring liver stiffness. Unfortunately, these diagnostic modalities did not have enough diagnostic accuracy. [24,25]

In conclusion, the diagnosis of NRH based on histopathology alone is unreliable. In case of discrepancy between clinical findings and histology, reports on the prevalence of NRH should be interpreted with caution. For the moment, our study stresses the importance of close collaboration between the clinician and the pathologist before drawing definitive clinical conclusions or taking any therapeutic decision solely based on the presence of NRH on a liver biopsy.
